# 1-Phenyl-3-(2,4,6-trimethoxy­phen­yl)prop-2-en-1-one

**DOI:** 10.1107/S1600536809041877

**Published:** 2009-10-23

**Authors:** Jianzhang Wu, Li Zhang, Jing Wang, Shulin Yang, Xiaokun Li

**Affiliations:** a Institute of Biotechnology, Nanjing University of Science and Technology, Nanjing, Jiangsu Province, 210094, People’s Republic of China; b School of Pharmacy, Wenzhou Medical College, Wenzhou, Zhejiang Province, 325035, People’s Republic of China

## Abstract

In the title compound, C_18_H_18_O_4_, a derivative of biologically active chalcones, the dihedral angle between the two rings is 7.43 (7)°. The molecule adopts an *E* configuration about the central olefinic bonds. In the crystal, there are no strong inter­actions between the mol­ecules.

## Related literature

For related structures, see: Subbiah Pandi *et al.* (2003 ▶); Low *et al.* (2002 ▶); Yathirajan *et al.* (2006 ▶); Suwunwong *et al.* (2009 ▶); Jasinski *et al.* (2009 ▶). For background to and applications of chalcones, see: Dimmock *et al.* (1999 ▶); Sivakumar *et al.* (2009 ▶); Echeverria *et al.* (2009 ▶); Kontogiorgis *et al.* (2008 ▶); Dominguez *et al.* (2005 ▶); Nowakowska (2007 ▶).
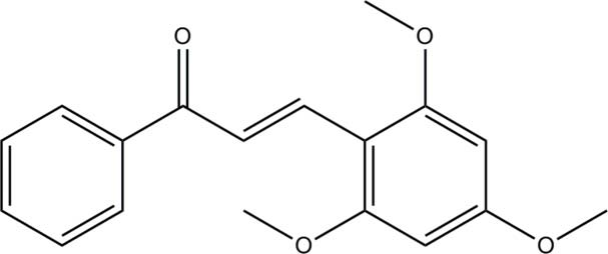


## Experimental

### 

#### Crystal data

C_18_H_18_O_4_
*M* *_r_* = 298.32
Monoclinic, 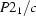

*a* = 9.0052 (10) Å
*b* = 14.9245 (17) Å
*c* = 11.7658 (14) Å
β = 104.315 (2)°
*V* = 1532.2 (3) Å^3^
*Z* = 4
Mo *K*α radiation
μ = 0.09 mm^−1^
*T* = 273 K
0.12 × 0.10 × 0.05 mm

#### Data collection

Bruker APEXII CCD area-detector diffractometer
Absorption correction: multi-scan (*SADABS*; Bruker, 2001 ▶) *T*_min_ = 0.989, *T*_max_ = 0.996
7895 measured reflections
2701 independent reflections
2125 reflections with *I* > 2σ(*I*)
*R*_int_ = 0.044

#### Refinement

*R*[*F* ^2^ > 2σ(*F* ^2^)] = 0.039
*wR*(*F* ^2^) = 0.132
*S* = 1.00
2701 reflections
203 parameters
H-atom parameters constrained
Δρ_max_ = 0.15 e Å^−3^
Δρ_min_ = −0.16 e Å^−3^

### 

Data collection: *APEX2* (Bruker, 2004 ▶); cell refinement: *SAINT-Plus* (Bruker, 2001 ▶); data reduction: *SAINT-Plus*; program(s) used to solve structure: *SHELXS97* (Sheldrick, 2008 ▶); program(s) used to refine structure: *SHELXL97* (Sheldrick, 2008 ▶); molecular graphics: *SHELXTL* (Sheldrick, 2008 ▶); software used to prepare material for publication: *SHELXTL*.

## Supplementary Material

Crystal structure: contains datablocks I, global. DOI: 10.1107/S1600536809041877/wn2350sup1.cif

Structure factors: contains datablocks I. DOI: 10.1107/S1600536809041877/wn2350Isup2.hkl

Additional supplementary materials:  crystallographic information; 3D view; checkCIF report

## Figures and Tables

**Fig. 1.**
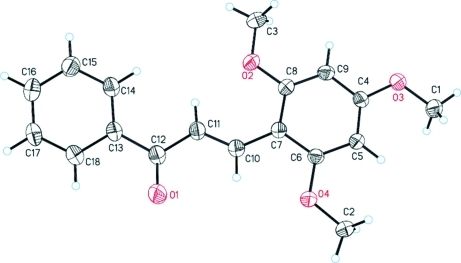
The molecular structure of the title compound, showing 30% displacement ellipsoids for the non-hydrogen atoms. Hydrogen atoms are drawn as spheres of arbitrary radius.

## supplementary crystallographic information

### Comment

Chalcones, which have the common skeleton of 1,3-diaryl-2-propen-1-ones, are natural products, distributed widely in fruits, vegetables etc. Natural and synthetical chalcones have wide-ranging biological properties, including antimicrobial, antifungal, antioxidant, antiangiogenic, antitumor and anti-inflammatory activities (Dimmock *et al.*, 1999; Sivakumar *et al.*, 2009; Echeverria *et al.*, 2009; Kontogiorgis *et al.*, 2008). The chalcone derivatives with trimethoxyphenyl substitution have also been reported to have a wide range of biological activities (Suwunwong *et al.*, 2009; Jasinski *et al.*, 2009; Dominguez *et al.*, 2005; Nowakowska, 2007).

The present investigation is a continuation of our broad program of work on the synthesis and structural study of chalcones and their derivatives. Investigation of these structures may be helpful in the design and synthesis of  new compounds. In order to understand the geometrical features and the underlying intermolecular interactions which hold the assembly of molecules in the crystal structure, an X-ray study of the title compound was carried out.

It is approximately planar and the dihedral angle between the two rings is 7.43 (7)°. The H atoms of the central propenone group are *trans*. The average value of the bond distances [1.385 (5) Å] and exocyclic bond angles [120.7 (4)°] in the benzene and phenyl rings have normal values which agree quite well with the values reported in the literature for some analogous structures (Subbiah Pandi *et al.*, 2003; Low *et al.*, 2002; Yathirajan *et al.*, 2006).

### Experimental

Acetophenone (15 mmol) was dissolved in ethanol (5 ml) and crushed KOH (15 mmol) was added. The flask was immersed in a bath of crushed ice and a solution of 2,4,6-trimethoxybenzaldehyde (15 mmol) in ethanol (5 mmol) was added. The reaction mixture was stirred at 300 K and completion of the reaction was monitored by thin-layer chromatography. Ice-cold water was added to the reaction mixture after 48 h and the yellow solid that separated was filtered off, washed with water and cold ethanol, dried and purified by column chromatography on silica gel (yield:68%). Single crystals of the title compound were grown in a CH_2_Cl_2_/CH_3_OH mixture (5:2 *v*/*v*) by slow evaporation (mp 436–437 K).

### Refinement

The H atoms were positioned geometrically (C—H = 0.93 and 0.96 Å) and refined as riding with *U*_iso_(H) = 1.2*U*_eq_(C) or 1.5*U*_eq_(methyl C).

### Crystal data

**Table:**

C_18_H_18_O_4_	*F*(000) = 632
*M**_r_* = 298.32	*D*_x_ = 1.293 Mg m^−^^3^
Monoclinic, *P*2_1_/*c*	Mo *K*α radiation, λ = 0.71073 Å
Hall symbol: -P 2ybc	Cell parameters from 3499 reflections
*a* = 9.0052 (10) Å	θ = 2.7–27.9°
*b* = 14.9245 (17) Å	µ = 0.09 mm^−^^1^
*c* = 11.7658 (14) Å	*T* = 273 K
β = 104.315 (2)°	Block, colorless
*V* = 1532.2 (3) Å^3^	0.12 × 0.10 × 0.05 mm
*Z* = 4	

### Data collection

**Table:**

Bruker APEXII CCD area-detector diffractometer	2701 independent reflections
Radiation source: fine-focus sealed tube	2125 reflections with *I* > 2σ(*I*)
graphite	*R*_int_ = 0.044
φ and ω scans	θ_max_ = 25.0°, θ_min_ = 2.3°
Absorption correction: multi-scan (*SADABS*; Bruker, 2001)	*h* = −10→10
*T*_min_ = 0.989, *T*_max_ = 0.996	*k* = −17→15
7895 measured reflections	*l* = −13→13

### Refinement

**Table:**

Refinement on *F*^2^	Secondary atom site location: difference Fourier map
Least-squares matrix: full	Hydrogen site location: inferred from neighbouring sites
*R*[*F*^2^ > 2σ(*F*^2^)] = 0.039	H-atom parameters constrained
*wR*(*F*^2^) = 0.132	*w* = 1/[σ^2^(*F*_o_^2^) + (0.0938*P*)^2^] where *P* = (*F*_o_^2^ + 2*F*_c_^2^)/3
*S* = 1.00	(Δ/σ)_max_ = 0.001
2701 reflections	Δρ_max_ = 0.14 e Å^−^^3^
203 parameters	Δρ_min_ = −0.16 e Å^−^^3^
0 restraints	Extinction correction: *SHELXL*, Fc^*^=kFc[1+0.001xFc^2^λ^3^/sin(2θ)]^-1/4^
Primary atom site location: structure-invariant direct methods	Extinction coefficient: 0.353 (18)

### Special details

**Table:**

Geometry. All e.s.d.'s (except the e.s.d. in the dihedral angle between two l.s. planes) are estimated using the full covariance matrix. The cell e.s.d.'s are taken into account individually in the estimation of e.s.d.'s in distances, angles and torsion angles; correlations between e.s.d.'s in cell parameters are only used when they are defined by crystal symmetry. An approximate (isotropic) treatment of cell e.s.d.'s is used for estimating e.s.d.'s involving l.s. planes.
Refinement. Refinement of *F*^2^ against ALL reflections. The weighted *R*-factor *wR* and goodness of fit *S* are based on *F*^2^, conventional *R*-factors *R* are based on *F*, with *F* set to zero for negative *F*^2^. The threshold expression of *F*^2^ > σ(*F*^2^) is used only for calculating *R*-factors(gt) *etc*. and is not relevant to the choice of reflections for refinement. *R*-factors based on *F*^2^ are statistically about twice as large as those based on *F*, and *R*- factors based on ALL data will be even larger.

### Fractional atomic coordinates and isotropic or equivalent isotropic displacement parameters (Å^2^)

**Table:**

	*x*	*y*	*z*	*U*_iso_*/*U*_eq_	
C1	0.9745 (2)	−0.05689 (12)	0.14050 (16)	0.0745 (5)	
H1A	1.0183	−0.0376	0.2196	0.112*	
H1B	1.0548	−0.0662	0.1012	0.112*	
H1C	0.9196	−0.1119	0.1415	0.112*	
C2	0.65289 (18)	−0.07567 (10)	0.42207 (13)	0.0599 (4)	
H2A	0.6579	−0.1285	0.3765	0.090*	
H2B	0.6153	−0.0913	0.4890	0.090*	
H2C	0.7534	−0.0500	0.4479	0.090*	
C3	0.44257 (19)	0.21153 (10)	−0.08152 (13)	0.0619 (4)	
H3A	0.5373	0.2441	−0.0605	0.093*	
H3B	0.3608	0.2517	−0.1166	0.093*	
H3C	0.4499	0.1652	−0.1364	0.093*	
C4	0.74716 (15)	0.02951 (9)	0.11985 (12)	0.0466 (4)	
C5	0.72022 (14)	−0.00525 (9)	0.22193 (11)	0.0462 (4)	
H5	0.7898	−0.0443	0.2687	0.055*	
C6	0.58735 (14)	0.01944 (9)	0.25291 (11)	0.0424 (4)	
C7	0.47895 (14)	0.07868 (8)	0.18520 (11)	0.0407 (4)	
C8	0.51526 (15)	0.11331 (9)	0.08332 (11)	0.0437 (4)	
C9	0.64562 (16)	0.08890 (9)	0.05100 (12)	0.0483 (4)	
H9	0.6658	0.1122	−0.0170	0.058*	
C10	0.34214 (15)	0.10052 (9)	0.22290 (12)	0.0456 (4)	
H10	0.3369	0.0743	0.2935	0.055*	
C11	0.22244 (15)	0.15177 (10)	0.17373 (12)	0.0509 (4)	
H11	0.2175	0.1775	0.1009	0.061*	
C12	0.09861 (15)	0.16858 (10)	0.23113 (12)	0.0512 (4)	
C13	−0.02269 (14)	0.23528 (9)	0.17775 (12)	0.0469 (4)	
C14	−0.01819 (17)	0.28712 (10)	0.08031 (13)	0.0557 (4)	
H14	0.0600	0.2784	0.0426	0.067*	
C15	−0.12880 (19)	0.35148 (11)	0.03909 (15)	0.0658 (5)	
H15	−0.1239	0.3863	−0.0255	0.079*	
C16	−0.24600 (18)	0.36410 (12)	0.09338 (15)	0.0665 (5)	
H16	−0.3197	0.4079	0.0661	0.080*	
C17	−0.25405 (17)	0.31221 (12)	0.18758 (15)	0.0650 (5)	
H17	−0.3346	0.3200	0.2232	0.078*	
C18	−0.14374 (16)	0.24862 (11)	0.22991 (13)	0.0568 (4)	
H18	−0.1501	0.2141	0.2943	0.068*	
O1	0.09525 (13)	0.13297 (9)	0.32420 (10)	0.0788 (4)	
O2	0.41260 (11)	0.17261 (7)	0.02032 (8)	0.0580 (3)	
O3	0.87208 (11)	0.01000 (7)	0.08015 (9)	0.0645 (4)	
O4	0.55259 (11)	−0.01253 (7)	0.35229 (9)	0.0568 (3)	

### Atomic displacement parameters (Å^2^)

**Table:**

	*U*^11^	*U*^22^	*U*^33^	*U*^12^	*U*^13^	*U*^23^
C1	0.0661 (10)	0.0857 (12)	0.0833 (11)	0.0327 (9)	0.0404 (9)	0.0290 (10)
C2	0.0601 (9)	0.0681 (10)	0.0523 (9)	0.0107 (8)	0.0156 (7)	0.0195 (7)
C3	0.0716 (10)	0.0603 (10)	0.0518 (9)	0.0047 (8)	0.0115 (8)	0.0158 (7)
C4	0.0438 (7)	0.0485 (8)	0.0512 (8)	0.0025 (6)	0.0186 (6)	0.0020 (6)
C5	0.0450 (8)	0.0464 (8)	0.0483 (8)	0.0063 (6)	0.0137 (6)	0.0071 (6)
C6	0.0441 (7)	0.0449 (7)	0.0390 (7)	−0.0012 (6)	0.0119 (6)	0.0025 (6)
C7	0.0407 (7)	0.0426 (7)	0.0384 (7)	0.0006 (5)	0.0088 (5)	−0.0029 (5)
C8	0.0455 (8)	0.0414 (7)	0.0421 (7)	0.0021 (6)	0.0067 (6)	0.0006 (6)
C9	0.0540 (8)	0.0510 (8)	0.0423 (8)	0.0014 (6)	0.0164 (6)	0.0065 (6)
C10	0.0455 (8)	0.0499 (8)	0.0409 (7)	0.0007 (6)	0.0097 (6)	−0.0048 (6)
C11	0.0463 (8)	0.0626 (9)	0.0430 (8)	0.0072 (6)	0.0097 (6)	−0.0019 (6)
C12	0.0445 (8)	0.0646 (9)	0.0433 (8)	0.0024 (7)	0.0086 (6)	−0.0059 (7)
C13	0.0392 (7)	0.0536 (8)	0.0460 (8)	−0.0003 (6)	0.0071 (6)	−0.0107 (6)
C14	0.0504 (8)	0.0632 (9)	0.0559 (9)	0.0055 (7)	0.0174 (7)	−0.0042 (7)
C15	0.0666 (10)	0.0660 (10)	0.0647 (10)	0.0118 (8)	0.0159 (8)	0.0051 (8)
C16	0.0532 (9)	0.0684 (11)	0.0751 (11)	0.0148 (8)	0.0107 (8)	−0.0029 (9)
C17	0.0444 (8)	0.0741 (11)	0.0801 (12)	0.0072 (7)	0.0221 (8)	−0.0086 (9)
C18	0.0496 (8)	0.0657 (9)	0.0580 (9)	−0.0010 (7)	0.0188 (7)	−0.0053 (7)
O1	0.0688 (8)	0.1132 (10)	0.0602 (7)	0.0294 (7)	0.0272 (6)	0.0240 (7)
O2	0.0577 (6)	0.0647 (7)	0.0522 (6)	0.0165 (5)	0.0150 (5)	0.0177 (5)
O3	0.0587 (7)	0.0769 (8)	0.0675 (7)	0.0206 (5)	0.0340 (6)	0.0224 (5)
O4	0.0533 (6)	0.0719 (7)	0.0500 (6)	0.0154 (5)	0.0216 (5)	0.0200 (5)

### Geometric parameters (Å, °)

**Table:**

C1—O3	1.4241 (17)	C8—O2	1.3596 (15)
C1—H1A	0.9600	C8—C9	1.3697 (19)
C1—H1B	0.9600	C9—H9	0.9300
C1—H1C	0.9600	C10—C11	1.3317 (19)
C2—O4	1.4182 (16)	C10—H10	0.9300
C2—H2A	0.9600	C11—C12	1.4611 (19)
C2—H2B	0.9600	C11—H11	0.9300
C2—H2C	0.9600	C12—O1	1.2244 (17)
C3—O2	1.4163 (17)	C12—C13	1.496 (2)
C3—H3A	0.9600	C13—C18	1.391 (2)
C3—H3B	0.9600	C13—C14	1.392 (2)
C3—H3C	0.9600	C14—C15	1.382 (2)
C4—O3	1.3521 (16)	C14—H14	0.9300
C4—C9	1.3827 (19)	C15—C16	1.375 (2)
C4—C5	1.3836 (19)	C15—H15	0.9300
C5—C6	1.3842 (17)	C16—C17	1.369 (2)
C5—H5	0.9300	C16—H16	0.9300
C6—O4	1.3688 (15)	C17—C18	1.375 (2)
C6—C7	1.4088 (18)	C17—H17	0.9300
C7—C8	1.4158 (18)	C18—H18	0.9300
C7—C10	1.4461 (18)		
			
O3—C1—H1A	109.5	C8—C9—C4	119.73 (12)
O3—C1—H1B	109.5	C8—C9—H9	120.1
H1A—C1—H1B	109.5	C4—C9—H9	120.1
O3—C1—H1C	109.5	C11—C10—C7	130.97 (13)
H1A—C1—H1C	109.5	C11—C10—H10	114.5
H1B—C1—H1C	109.5	C7—C10—H10	114.5
O4—C2—H2A	109.5	C10—C11—C12	121.74 (13)
O4—C2—H2B	109.5	C10—C11—H11	119.1
H2A—C2—H2B	109.5	C12—C11—H11	119.1
O4—C2—H2C	109.5	O1—C12—C11	121.96 (13)
H2A—C2—H2C	109.5	O1—C12—C13	119.16 (13)
H2B—C2—H2C	109.5	C11—C12—C13	118.79 (13)
O2—C3—H3A	109.5	C18—C13—C14	117.99 (13)
O2—C3—H3B	109.5	C18—C13—C12	118.71 (13)
H3A—C3—H3B	109.5	C14—C13—C12	123.26 (12)
O2—C3—H3C	109.5	C15—C14—C13	120.66 (14)
H3A—C3—H3C	109.5	C15—C14—H14	119.7
H3B—C3—H3C	109.5	C13—C14—H14	119.7
O3—C4—C9	114.95 (12)	C14—C15—C16	120.09 (16)
O3—C4—C5	123.95 (12)	C14—C15—H15	120.0
C9—C4—C5	121.09 (12)	C16—C15—H15	120.0
C6—C5—C4	118.50 (12)	C15—C16—C17	119.95 (15)
C6—C5—H5	120.7	C15—C16—H16	120.0
C4—C5—H5	120.7	C17—C16—H16	120.0
O4—C6—C5	121.82 (11)	C16—C17—C18	120.38 (14)
O4—C6—C7	115.41 (11)	C16—C17—H17	119.8
C5—C6—C7	122.77 (11)	C18—C17—H17	119.8
C6—C7—C8	115.79 (11)	C13—C18—C17	120.91 (15)
C6—C7—C10	119.45 (11)	C13—C18—H18	119.6
C8—C7—C10	124.75 (12)	C17—C18—H18	119.5
O2—C8—C9	122.39 (12)	C8—O2—C3	118.82 (11)
O2—C8—C7	115.53 (11)	C4—O3—C1	118.07 (11)
C9—C8—C7	122.08 (12)	C6—O4—C2	118.61 (10)

## References

[bb1] Bruker (2001). *SADABS and *SAINT-Plus** Bruker AXS Inc., Madison, Wisconsin, USA.

[bb2] Bruker (2004). *APEX2* Bruker AXS Inc., Madison, Wisconsin, USA.

[bb3] Dimmock, J. R., Elias, D. W., Beazely, M. A. & Kandepu, N. M. (1999). *Curr. Med. Chem.* **6**, 1125–1150. 10519918

[bb4] Dominguez, J. N., Leon, C., Rodrigues, J., Gamboa de Dominguez, N., Gut, J. & Rosenthal, P. J. (2005). *Farmaco.* **60**, 307–311. 10.1016/j.farmac.2005.01.005 15848205

[bb5] Echeverria, C., Santibanez, J. F., Donoso-Tauda, O., Escobar, C. A. & Ramirez-Tagle, R. (2009). *Int. J. Mol. Sci.* **10**, 221–31. 10.3390/ijms10010221 PMC2662465 19333443

[bb6] Jasinski, J. P., Butcher, R. J., Veena, K., Narayana, B. & Yathirajan, H. S. (2009). *Acta Cryst.* E**65**, o1965–o1966.

[bb7] Kontogiorgis, C., Mantzanidou, M. & Hadjipavlou-Litina, D. (2008). *Mini Rev. Med. Chem.* **8**, 1224–1242. 10.2174/138955708786141034 18855737

[bb8] Low, J. N., Cobo, J., Nogueras, M., Sánchez, A., Albornoz, A. & Abonia, R. (2002). *Acta Cryst.* C**58**, o42–o45. 10.1107/s0108270101018297 11781492

[bb9] Nowakowska, Z. (2007). *Eur. J. Med. Chem.* **42**, 125–137. 10.1016/j.ejmech.2006.09.019 17112640

[bb10] Sheldrick, G. M. (2008). *Acta Cryst.* A**64**, 112–122. 10.1107/S0108767307043930 18156677

[bb11] Sivakumar, P. M., Muthu Kumar, T. & Doble, M. (2009). *Chem. Biol. Drug Des.* **4**, 68–79. 10.1111/j.1747-0285.2009.00828.x 19519746

[bb12] Subbiah Pandi, A., Velmurugan, D., Shanmuga Sundara Raj, S., Fun, H.-K. & Bansal, M. C. (2003). *Acta Cryst.* C**59**, o302–o304. 10.1107/s0108270103008679 12794345

[bb13] Suwunwong, T., Chantrapromma, S., Pakdeevanich, P. & Fun, H.-K. (2009). *Acta Cryst.* E**65**, o1575–o1576. 10.1107/S1600536809021850 PMC2969210 21582853

[bb14] Yathirajan, H. S., Sarojini, B. K., Narayana, B., Bindya, S. & Bolte, M. (2006). *Acta Cryst.* E**62**, o3629–o3630.

